# An ophthalmologist with myocardial bridging developed takotsubo cardiomyopathy while operating; A case report and literature review

**DOI:** 10.1002/ccr3.7353

**Published:** 2023-11-22

**Authors:** Anas Al‐sadi, Awad Al‐Qahtani, Osama Al‐Khalaila, Israa Jawarneh, Sabir Abdul Karim

**Affiliations:** ^1^ Department of Internal Medicine Hamad medical corporation Doha Qatar; ^2^ Department of Cardiology, Heart Hospital Hamad Medical Corporation Doha Qatar; ^3^ Department of Internal Medicine King Abdullah University Hospital Ar Ramtha Jordan

**Keywords:** health care, myocardial bridging, reversible cardiomyopathy, stress, takotsubo cardiomyopathy, workers

## Abstract

**Key Clinical Message:**

Healthcare workers are prone to very high level of physical as well as emotional stress that lead to devastating health‐related consequences which include but not limited to cardiovascular events that may lead to death. Recognizing the risk of Takotsubo Cardiomyopathy among healthcare worker is the main aim of this report.

**Abstract:**

Takotsubo Cardiomyopathy (TC) is a reversible left ventricular wall motion abnormality that could not be explained by coronary artery disease and is typically precipitated by either emotional or physical stress. There is no sufficient data regarding the incidence of TC among healthcare workers and people with myocardial bridging. Here we are describing a case of an ophthalmologist with myocardial bridging who developed TC while in the operation theater.

## 1 INTRODUCTION

The impact of occupational stress on physical and mental health is a serious challenge for workers and especially healthcare workers given their long working hours and emotional demands. Takotsubo Cardiomyopathy (TC) is a multifactorial disease where stressors play a pivotal role in its pathogenesis. The relation between myocardial bridging (MB) and TC has no sufficient data. Initial presentation of TC is often confused with acute coronary syndrome and patients typically required evaluation with cardiac catheterization and cardiac imaging to reach the diagnosis. Severe acute complications in TC are similar to patients with acute coronary syndrome, however, the prognosis in long term is good with full recovery within weeks.

## 2 CASE PRESENTATION

Our patient is a 63‐year‐old Ophthalmologist, known to have supraventricular tachycardia (SVT) well controlled by flecainide for 10 years and newly diagnosed dyslipidemia. She was doing an ophthalmologic surgery when she suddenly experienced retrosternal heaviness, that radiated to her left shoulder associated with dizziness without shortness of breath, palpitation, or altered level of consciousness. On further questioning, patient admit that she had a milder attack of similar pain 1 week ago but subsided spontaneously within 5 min. She denied any exertional shortness of breath, orthopnea, paroxysmal nocturnal dyspnea, or lower limb edema.

She is a senior ophthalmologist who came as a visiting professor to Qatar from Canada on a long‐haul flight journey a few days before the event and she could not sleep well over the past few days because of the travel and jet lag. On the day of the event, she described that she has been extremely stressed as she had a very busy schedule; she started her day by giving lectures to medical students and residents, then she went to the operation theater where she had a long list of complicated eye surgeries.

Upon presentation to the emergency room, she was pain‐free, her vitals were normal apart from mild tachycardia (88/min). Her body mass index (BMI) was 22. Jugular venous pressure was not elevated and her heart, lung, and abdominal examinations were unremarkable and no lower limb edema.

**FIGURE 1:**
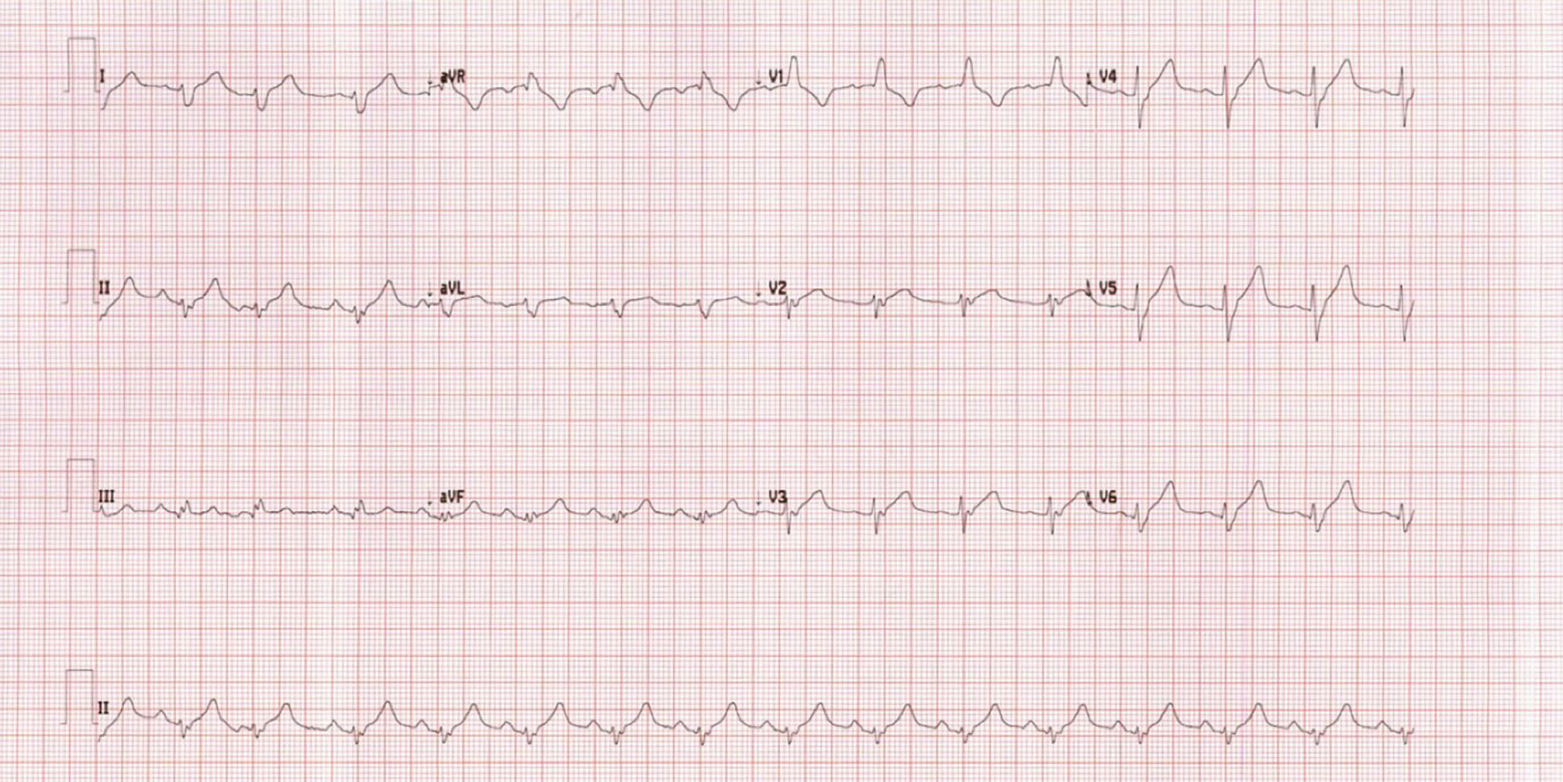
initial ECG showed RBBB and right axis deviation.

Her ECG showed sinus rhythm with right bundle branch block (RBBB) and right axis deviation (see Figure 1). There were no previous ECGs to compare but according to the patient she had these findings before.

Serial troponin level increased from 414 ng/L (normal 3–10 ng/L) to 567 ng/L then decreased slowly to 34 ng/L. her complete blood count, renal, electrolytes, and liver functions all were unremarkable. The lipid panel was as follows: total cholesterol 8.5 mmoL/L, LDL 6.2 mmoL/L, Triglycerides 0.7 mmoL/L, and HDL 2 mmoL/L. Her HbA1c was 5.6% and her thyroid function was normal.

She was admitted to CCU as a case of high‐risk non‐ST segment elevation acute coronary syndrome versus TC and she was started on appropriate treatment. Flecainide was replaced with a calcium channel blocker.

**FIGURE 2:**
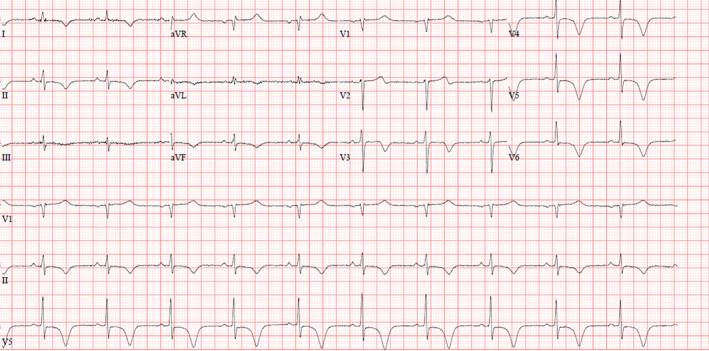
ECG on next day showed total resolution of previous abnormalities with some non‐specific ST‐T changes in anterolateral leads.

The next day, her ECG showed a resolution of previous findings noted on admission with the development of deep T wave inversion in anterolateral and inferior leads (see Figure 2).

**FIGURE 3:**
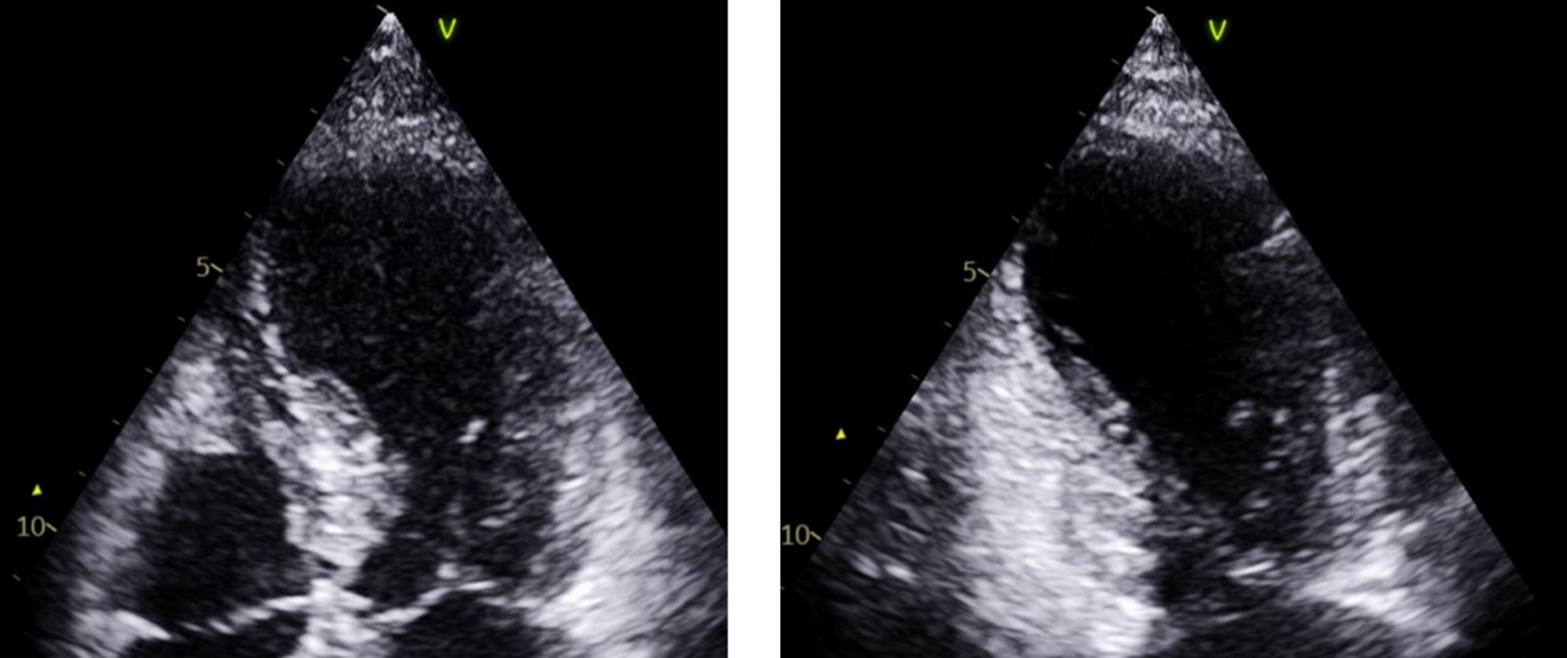
Transthoracic echocardiography apical 4‐chamber (A) and 2‐chamber (B) views at end‐systole showing apical akinesia.

Her echocardiogram showed an ejection fraction of 47% with apical akinesia making TC more likely than acute coronary syndrome (see Figure 3).

**FIGURE 4:**
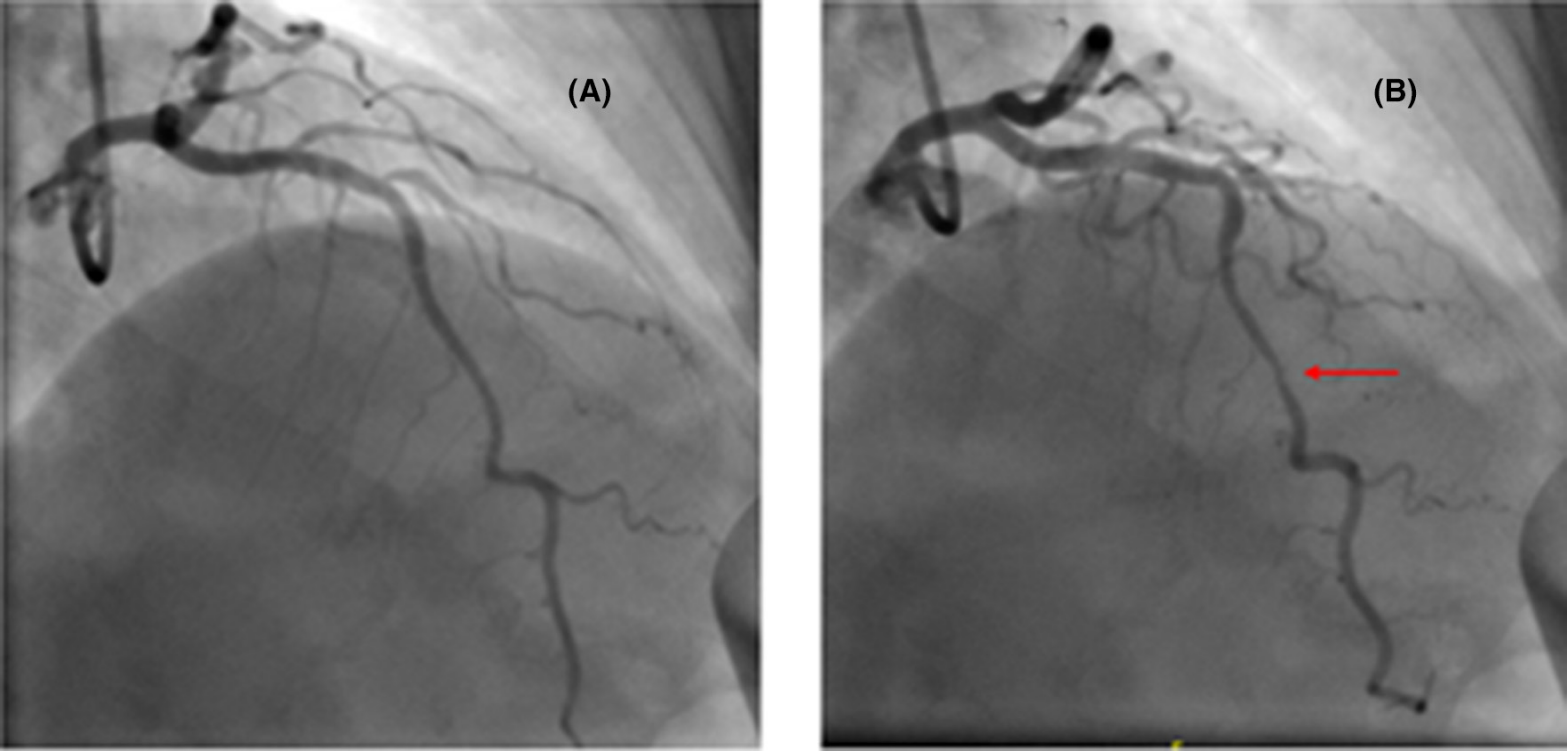
(A) Coronary angiogram left‐anterior oblique (LAO)—cranial view at end‐diastole shows normal left‐main coronary and left anterior descending artery (LAD) arteries. (B); Coronary angiogram LAO—cranial view at end‐systole shows a short segment of moderate narrowing at distal LAD artery (red‐arrow) suggestive of myocardial bridging.

Coronary angiogram showed normal coronaries apart from moderate MB in the distal left anterior descending artery (LAD) (see Figure 4).

**FIGURE 5:**
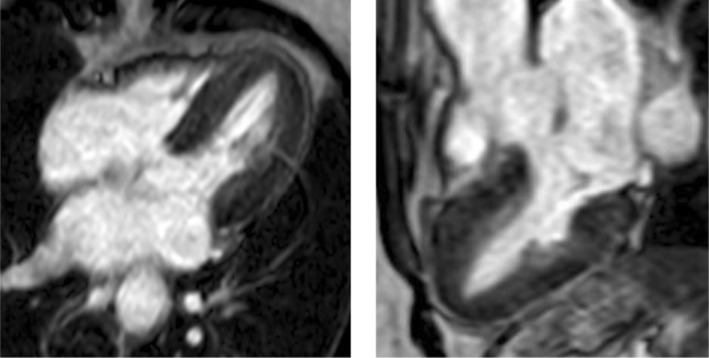
Delayed gadolinium sequences acquired in three‐chamber and four‐chamber views 10 min after gadolinium injection shows no enhancement.

Cardiac magnetic resonance (CMR) images were obtained on a 1.5 Tesla (Philips Ingenia) scanner and findings from the steady‐state free precession (SSFP) cines showed normal biventricular volumes and function. (LVEF 75%, RVEF 57%). The parametric native T1 mapping sequence demonstrated significantly raised values ranging between 1083 and 1171 ms (normal range 965–1035). On post‐gadolinium administration, early and late acquisition (see Figure 5), there was no enhancements (LGE) to suggest any infarction or myocardial necrosis.

Combining the patient's history, quick recovery of the left ventricular myocardial contractility and the CMR evidence of elevated native T1 mapping values, the overall features are suggestive of stress‐induced TC.

**FIGURE 6:**
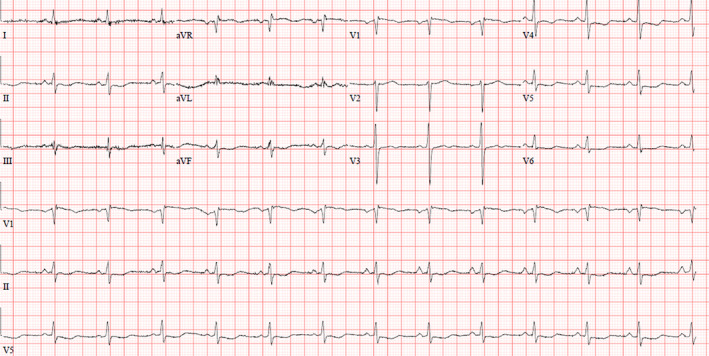
ECG on the day of discharge showed non‐specific ST‐T changes.

Throughout her hospital course, the patient remained asymptomatic. Her ECG on the day of discharge showed non‐specific ST‐T changes (see Figure 6).

## 3 DISCUSSION

TC, also known as stress‐induced cardiomyopathy, and broken heart syndrome, was first described in Japan in 1990.^1^ Minhas et al. and Murugiah et al. showed a significant increase in the incidence and hospitalization rate of TC due to increasing awareness and early access to invasive coronary angiogram.^2^, ^3^ TC is characterized by reversible regional left ventricular (LV) wall motion abnormalities that occur primarily after a recent stressful condition.^4^ Although emotional stress, such as the death of a family member, was classically considered a precipitating factor, physical stressors are now recognized to be more common.^5^ The disease is by far more common in females than males with a female percentage reaching 90% in The International Takotsubo Registry which was done across 26 centers in Europe and the United States with a median age of 66.8 years.^6^ It has been reported in a variety of races, but it is uncommon in Hispanics and African Americans.^7^

The exact pathophysiology of TC is not well understood, but it seems to be the result of interaction between multiple factors including catecholamine myocardial toxicity, autonomic nervous dysfunction, and multivessel coronary artery spasm.^8^

The usual clinical presentation typically mimics acute coronary syndrome with chest pain being the most common symptom,^6^ along with ECG changes and rising cardiac biomarkers.^9^ Cardiac catheterization typically shows normal coronary arteries. Echocardiogram typically shows apical ballooning of the left ventricle with akinetic or hypokinetic apical segments and hyperdynamic basal segments in almost 80% of cases.^6^, ^10^ Other atypical variants include involvement of the right ventricle, LV basement segments (also known as reverse Takotsubo), or LV mid‐cavity also been described.^11^

The revised Mayo clinic diagnostic criteria to diagnose TC include the followings: Transient dyskinesia of left ventricular midsegment, regional wall motion abnormalities beyond a single epicardial vascular distribution, absence of obstructive coronary artery disease, new ECG changes or modest troponin elevation, and absence of pheochromocytoma and myocarditis.^12^

The overall prognosis for patients who survive the acute event is usually good with full recovery of systolic ventricular function within 1–4 weeks.^13^ However, the risk of severe in‐hospital complications such as cardiogenic shock, the use of invasive or non‐invasive ventilation, cardiopulmonary resuscitation, and death is similar to patients with acute coronary syndrome.^6^

Our patient is a post‐menopausal female ophthalmologist who developed a typical clinical and radiological course of TC triggered by an unusually stressful schedule in the operation room.

The negative impact of occupational stress on physical and mental health is well‐recognized.^14^ Cardiovascular diseases have been linked with work stress across multiple demographic groups. One meta‐analysis showed a 50% excess risk of coronary heart disease among employees with work stress.^15^ Healthcare workers are especially exposed to highly stressful conditions given the long working hours, shift work, and emotional demands.^16^ Females are significantly at higher risk than males for stress among healthcare workers due to low social support.^17^ Given the association between stress and TC, healthcare workers may be at an increased risk for the disease, however, there is no sufficient data available regarding the incidence of TC in healthcare workers in general and doctors in particular. Only one case of TC in a healthcare worker was reported.^18^ To the date of writing this article, there were no reported cases published in the Pubmed database of TC triggered in a physician during duty hours.

There are conflicting published data regarding the association between MB and TC. In a study that included 42 patients with TC and 401 controls, MB was significantly more prevalent in the TC group (76% vs. 31%, *p* < 0.001).^19^ However, in two larger studies, MB prevalence was not significantly higher in patients with TC.^20^, ^21^

## 4 CONCLUSION

Physicians, especially surgeons, may be at increased risk of developing TC, and more effort should be directed to decrease their stressful work conditions, whether these were physical or emotional. MB could play a role in TC pathogenesis; however, more studies are needed to establish this association.

## AUTHOR CONTRIBUTIONS

**Anas Al‐sadi:** Validation; writing – original draft; writing – review and editing. **Awad Alqahtani:** Investigation; resources; validation. **Osama Alkhalaila:** Investigation; resources; writing – review and editing. **Israa Jawarneh:** Data curation; writing – review and editing. **Sabir Abdulkarim:** Investigation; resources.

## FUNDING INFORMATION

Open access funding provided by the Qatar National Library (QNL).

## CONFLICT OF INTEREST STATEMENT

No conflict of interest to be declared.

### CONSENT STATEMENT

Written informed consent was obtained from the patient to publish this report following the journal's patient consent policy.

## Data Availability

Data sharing is not applicable to this article as no new data were created or analyzed in this study.
